# A global metabolomics minefield: Confounding effects of preanalytical factors when studying rare disorders

**DOI:** 10.1002/ansa.202300010

**Published:** 2023-07-21

**Authors:** Hanne Bendiksen Skogvold, Steven Ray Haakon Wilson, Per Ola Rønning, Linda Ferrante, Siri Hauge Opdal, Torleiv Ole Rognum, Helge Rootwelt, Katja Benedikte Prestø Elgstøen

**Affiliations:** ^1^ Department of Mechanical, Electronic and Chemical Engineering, Faculty of Technology, Art and Design Oslo Metropolitan University Oslo Norway; ^2^ Department of Medical Biochemistry Oslo University Hospital Oslo Norway; ^3^ Department of Chemistry University of Oslo Oslo Norway; ^4^ Hybrid Technology Hub‐Centre of Excellence, Institute of Basic Medical Sciences, Faculty of Medicine University of Oslo Oslo Norway; ^5^ Department of Forensic Sciences, Section of Forensic Pathology and Clinical Forensic Medicine Oslo University Hospital Oslo Norway; ^6^ Department of Forensic Medicine Oslo University Hospital Oslo Norway

**Keywords:** dried blood spots, metabolomics, preanalytical effects, preanalytical variation, rare disorders, storage, sudden infant death syndrome

## Abstract

A common challenge when studying rare diseases or medical conditions is the limited number of patients, usually resulting in long inclusion periods as well as unequal sampling and storage conditions. The main purpose of this study was to demonstrate the challenges when comparing samples subject to different preanalytical conditions. We performed a global (commonly referred to as “untargeted”) liquid chromatography‐high resolution mass spectrometry metabolomics analysis of blood samples from cases of sudden infant death syndrome and controls stored as dried blood spots on a chemical‐free filter card for 15 years at room temperature compared with the same blood samples stored as whole blood at −80°C before preparing new dried blood spots using a chemically treated filter card. Principal component analysis plots distinctly separated the samples based on the type of filter card and storage, but not sudden infant death syndrome versus controls. Note that, 1263 out of 5161 and 642 out of 1587 metabolite features detected in positive and negative ionization mode, respectively, were found to have significant 2‐fold changes in amounts corresponding to different preanalytical conditions. The study demonstrates that the dried blood spot metabolome is largely affected by preanalytical factors. This emphasizes the importance of thoroughly addressing preanalytical factors during study design and interpretation, enabling identification of real, biological differences between sample groups whilst preventing other factors or random variation to be falsely interpreted as positive results.

**Abbreviations**
DBS: Dried blood spots
EDTA: Ethylenediaminetetraacetic acid
FTA: Flinders Technology Associates
Hct: Hematocrit
LC‐HRMS: Liquid chromatography‐high resolution mass spectrometry
LC‐MS: Liquid chromatography‐mass spectrometry
MS/MS: tandem mass spectrometry
PC: Principal component
PCA: Principal component analysis
SIDS: Sudden infant death syndrome
UHPLC: Ultra‐high performance liquid chromatography

## 1 INTRODUCTION

Metabolomics is the study of all metabolites in a sample, i.e., small molecules typically having a molecular weight < 1500 Da.^1^, ^2^ The total composition of metabolites in a sample is termed the metabolome. It is dynamic and differs over time and between cells, tissues, organs and body fluids in the organism.^1^, ^2^ For a human being, these metabolites are typically substrates, intermediates, and end‐products from all the thousands of biochemical reactions continuously taking place. Metabolites may also originate from diet, medications, etc., as well as the microbiomes of our intestinal tract, airways or other body surfaces.^3^ Furthermore, the metabolome is dependent on the physiological status, like physical activity and activities of daily life, as well as mirroring the biochemical status of health and disease.

Global metabolomics involves untargeted analyses of as many compounds as possible within a certain mass range and with the methodological conditions applied.^4^, ^5^ This approach enables the discovery of new and/or unexpected findings, e.g., identification of metabolites suitable as biomarkers for diagnosis, monitoring of natural disease progression and therapeutic response, as well as for revealing non‐compliance/adherence to treatment. Liquid chromatography‐high resolution mass spectrometry (LC‐HRMS) is the most commonly used technique for global metabolomics, as thousands of compounds can be detected in a single analysis with a high degree of sensitivity and selectivity.^4^ LC‐HRMS metabolomics is readily compatible with dried blood spot (DBS) analysis.^6^

A common challenge when studying rare diseases or medical conditions (i.e., ≤1 per 2000^7^) is the limited number of patients and samples available for analysis. The limited number of patients often necessitates the collection of samples over the course of several years and often from different medical centres. Inevitably, this often results in a multitude of differences in preanalytical factors such as variations in sample handling, storage conditions and time, and usually a lack of appropriately matched controls collected at the same time using identical procedures minimizing preanalytical differences. Even a minor degree of degradation or modifications of only a fraction of the metabolites caused by preanalytical differences will affect biochemical profiles and the overall global metabolome, impacting the possibility of correctly describing the biological status at the time of sampling and detecting true differences between cases and controls.^8^ Several studies have therefore focused on preanalytical effects on biological specimens, demonstrating how metabolite amounts change to varying degrees as a consequence of, e.g., storage conditions and storage time.^6^, ^9^, ^10^, ^11^, ^12^, ^13^, ^14^, ^15^, ^16^

The hematocrit (Hct) effect is a common challenge with DBS analysis. Hct is the volume percentage of red blood cells in whole blood. A higher Hct makes a blood sample more viscous, thereby affecting the spreading of the blood on the filter card, as well as increasing the overall concentration of predominantly intracellularly located metabolites in the sample, and reducing the amount of the metabolites primarily found in the plasma. Hct thus can affect both the recovery and the quantification of metabolites in DBS analysis.^17^

A case in point of a rare condition that often requires the collection of samples over the course of many years and thereby is subject to possible variations in preanalytical factors is sudden infant death syndrome (SIDS). SIDS is defined as the unexpected death of an infant less than 1 year of age, with the onset of the fatal episode apparently occurring during sleep, that remains unexplained after a thorough investigation, including the performance of a complete autopsy and review of the circumstances of death and the clinical history.^18^ While SIDS in the 1980s in Norway was the dominating cause of death in the post‐neonatal period with a rate of 2.4 deaths per 1000 live births, the condition is now rare due to the risk‐reducing campaigns introduced around 1990. SIDS now occurs in < 0.2 deaths per 1000 live births.^19^ Only 6 cases of SIDS were reported in Norway in 2021^20^ in a population with 56060 live births.^21^

Using our global metabolomics platform, we have performed a metabolomics analysis of blood samples obtained from autopsy during 1989−2003 from cases of SIDS and controls initially stored at −80°C. The SIDS and control samples were thawed both in 2003 and 2018, and DBS were prepared at both time points from the same blood sample tubes. Our global (often referred to as “untargeted”) metabolomics method covers a wide range of metabolites regarding polarity and chemical properties. The method has been demonstrated to have excellent retention time‐ and peak area repeatability, linearity, and ability to differentiate between groups by identifying discriminating metabolites, with a run time of 32.5 min.^22^ We initially wanted to investigate if we could detect differences in the DBS metabolome between cases of SIDS and controls that could be used as biomarkers of SIDS. In addition, we wanted to investigate the differences introduced by different storage times and storage conditions. However, this original investigation became moot because of preanalytical confounding effects as the samples were applied to two different types of filter cards. We here describe our analysis of these rare samples and our assessments of preanalytical factors. The aim of this communication is to highlight the importance of preanalytical factors and strict routines to reduce the detrimental effects of preanalytical variation on the analysis of rare diseases and conditions and on the interpretation of the results and the evaluation of the biological implications.

## 2 MATERIALS AND METHODS

### 2.1 Consumables and small equipment

Microtubes were from Sarstedt, and the thermomixer used was a Thermomixer comfort (Eppendorf). Filter cards were 903 Protein saver and Flinders Technology Associates (FTA) micro cards, both from Whatman (Cytiva) and PerkinElmer 226 cards. The LC column was a Pursuit XRs Diphenyl (Agilent Technologies) (250 × 2.0 mm, particle size 3 µm).

### 2.2 Chemicals

The water used was of type 1 (>18 MΩ cm) from Milli‐Q ultrapure water purification system (Merck Millipore). LC‐MS grade methanol was from Rathburn Chemicals . Formic acid (98%) was from Merck.

### 2.3 Subjects, samples and sample preparation

For a study comparing three types of filter cards (PerkinElmer 226, 903 Protein saver and FTA cards), we sampled DBS from three healthy volunteers on all three. The volunteers were of the same sex and of similar age. The samples were left to dry at room temperature for 4 h before preparation and analysis.

Metabolomics analyses of blood samples from healthy volunteers and samples from the SIDS biobank at Oslo University Hospital were approved by the Regional Committee for Medical and Health Research Ethics (REK) (REK 173346 and 2015/684). All healthy volunteers gave their informed consent prior to being included in the study. The study conforms to the Declaration of Helsinki.

The SIDS biobank at Oslo University Hospital was founded in 1984 and contains samples from approximately 800 cases. According to the research protocol for sampling cases of SIDS, the body fluids; blood, cerebrospinal fluid, vitreous humour, bile, urine (when present) and tissue specimens from all organs were sampled during the autopsy and stored. All samples were stored at −80°C.

The SIDS biobank is reviewed and approved by the National Committees for Research Ethics in Norway (REK 5980). All included cases are deceased and <4 years of age. All next of kin have received a letter giving information about autopsy and sampling for research, and were at the same time given the opportunity to register in a non‐research exclusion register. The next of kin were also explicitly informed that they could withdraw their consent at any time. None of the next of kin of the cases of SIDS and controls included in the present study have done so.

Whole blood samples from nine SIDS victims and three controls, collected during autopsy at Oslo University Hospital from 1989 to 2003, were used. The control samples included samples from infants who died a sudden traumatic death (Table 1). All samples were collected during autopsy and kept in a freezer at −80°C, with EDTA as an anticoagulant. The samples were thawed and pipetted to a filter card at two time intervals; the second time being 15 years later than the first. Thus, the storage conditions (time and temperature) of the two DBS samples from each case differed, as well as one extra freeze‐thaw cycle for the last time point.

**TABLE 1:** Diagnostic group, cause of death and storage time at −80°C from sampling during autopsy to pipetting to filter cards.

Diagnostic group	Cause of death	Time in years between sampling and pipetting to PerkinElmer 226 card (stored at −80°C)	Time in years between sampling and pipetting to FTA micro card (stored at −80°C)
SIDS	SIDS	14	29
SIDS	SIDS	13	28
SIDS	SIDS	11	26
SIDS	SIDS	10	25
SIDS	SIDS	9	24
SIDS	SIDS	5	20
SIDS	SIDS	4	19
SIDS	SIDS	3	18
SIDS	SIDS	2	17
Control	Violent death	5	20
Control	Violent death	2	17
Control	Violent death	0	15

**FIGURE 1:**
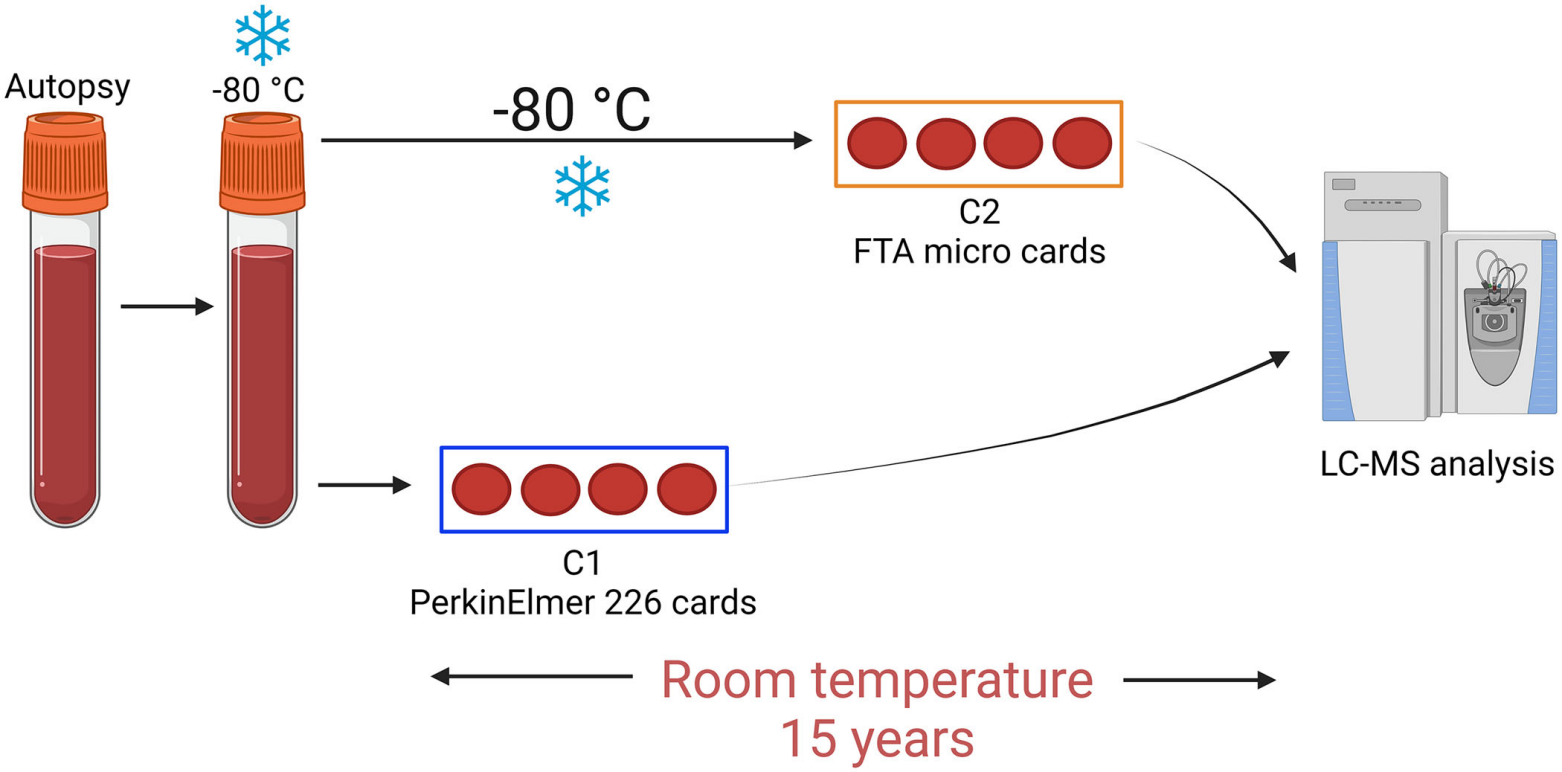
Illustration of the storage of the two dried blood spot samples from each case. C1; Blood prepared and stored as dried blood spot (DBS) (PerkinElmer 226 cards) for 15 years at room temperature, and C2; the same blood samples stored at −80°C for the same 15 years before preparation as DBS (Flinders Technology Associates [FTA] micro cards) shortly before analysis. Created with BioRender.com.

The DBS samples stored for 15 years without desiccant in envelopes at room temperature (“C1”) were compared with the same whole blood samples stored in a freezer at −80°C, thawed and prepared as DBS shortly before analysis (“C2”). Filter cards used for C1 were chemical‐free newborn screening cards (PerkinElmer 226), whereas, for C2, FTA micro cards (chemically treated to ensure protection of DNA) had been used. **Figure** **1** illustrates the preparation and storage of the two DBS samples from each case.

One punch from the centre of each DBS (∼3.2 mm spot size, equivalent to ∼3 µl whole blood) was extracted with 100 μl 80% aqueous methanol with 0.1% formic acid in a microtube. Extraction was performed using a thermomixer at 45°C for 45 min (700 rpm) before the samples were transferred to vials for analysis.

### 2.4 LC‐HRMS analysis

Full MS analyses were performed using our in‐house developed LC‐HRMS method,^22^ with a Dionex Ultimate 3000 UHPLC system and a Q Exactive Orbitrap with electrospray ionization (Thermo Fisher Scientific). Settings used in the MS/MS analyses are shown in Tables S1 and S2 in the supporting information (settings not described in the tables were the same as in Skogvold et al.,^22^ including LC and electrospray ionization settings). Samples were analyzed in both positive and negative ionization mode in separate injections. The samples in the pilot study comparing three types of filter cards were prepared and analyzed in the same batch. All C1 and C2 samples were prepared and analyzed together in a separate batch. C1 and C2 samples were injected every other time in the analysis sequence.

### 2.5 Interpretation tools and computer software

For metabolomics analyses, we used Xcalibur (version 4.0), Tune (version 2.8), and Chromeleon Xpress (version 6.80), all from Thermo Fisher Scientific. To evaluate the results on a global level, i.e., investigating potential differences of the metabolomes of the groups, principal component analysis (PCA) plots were used. For evaluation of the results on an individual metabolite level, volcano plots and box plots (based on the compound's peak areas) were used. Compound Discoverer 3.3 (Thermo Fisher Scientific) was used for data processing and interpretation, including the generation of PCA, volcano and box plots. The workflow “Untargeted Metabolomics with Statistics Detect Unknowns with ID using Online Databases and mzLogic” was used for data processing. For identification, the mass tolerance was 5 ppm and the retention time tolerance was 2 min. The minimum peak intensity was 200,000, and the chromatographic S/N threshold was 1.5.

## 3 RESULTS

### 3.1 Clear differences between filter cards in PCA plots

**FIGURE 2:**
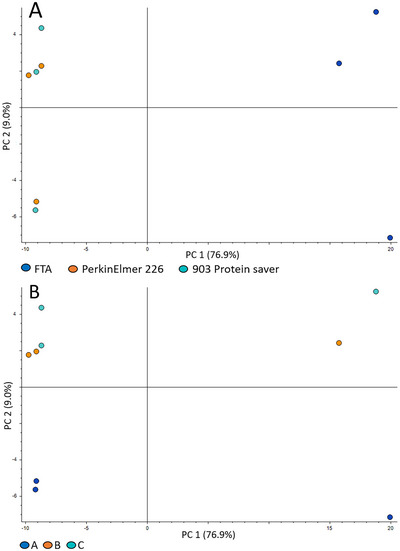
Principal component analysis (PCA) plots showing a clear separation between chemical‐free (903 Protein saver and PerkinElmer 226) and chemically treated (Flinders Technology Associates [FTA]) cards. (A) Colour by filter card. (B) Colour by person. One point represents one sample.

We compared three types of filter cards: PerkinElmer 226 and 903 Protein saver (both chemical‐free) and FTA micro cards (treated with chemicals to ensure the protection of DNA). When coloured based on filter card type, the two chemical‐free cards clustered together away from the FTA cards along principal component (PC) 1 (Figure 2A). When coloured based on person, persons B and C clustered together along PC 2, away from person A (Figure 2B). Comparing the two PCA plots unveils that the main factor contributing to differentiating the samples is the filter card type, not biological differences between the three healthy volunteers. PCA plots from the negative ionization mode analysis, for which PC 1 explains 76.9% of the variation, are shown. The same was observed for the positive ionization mode.

For positive and negative ionization modes, respectively, 4728 and 1506 metabolite features were detected. Different ions can be generated from a neutral metabolite in the electrospray ionization process, due to adduct formation, etc. These variations of a metabolite are referred to as “metabolite features”. Although they have identical retention times, during data processing the features may be perceived as different metabolites as the *m/z* is different. As such, the number of detected metabolite features is typically higher than the number of detected metabolites. Of the total number of detected metabolite features, 112 (positive ionization mode) and 138 (negative ionization mode) met the criteria of being significantly altered with a fold change of > 2 or < 0.5 and a p‐value < 0.05 when comparing the FTA card with the PerkinElmer 226 card. In positive and negative ionization mode, respectively, 63 and 118 metabolite features were detected with a larger peak area (i.e., fold change > 2) in FTA cards whereas 49 and 20 were detected with smaller peak areas (i.e., fold change < 0.5) compared to PerkinElmer 226 cards. This demonstrates that a substantial proportion (around 5% on average for positive and negative ionization) of the metabolites covered by our analysis was significantly altered when comparing FTA cards with chemical‐free filter cards. These results show that whether or not a filter card is chemically treated markedly affects the metabolites covered by our analysis, as all other preanalytical factors in this pilot study were identical.

### 3.2 Total ion chromatograms of DBS on different types of filter cards differ markedly from each other

**FIGURE 3:**
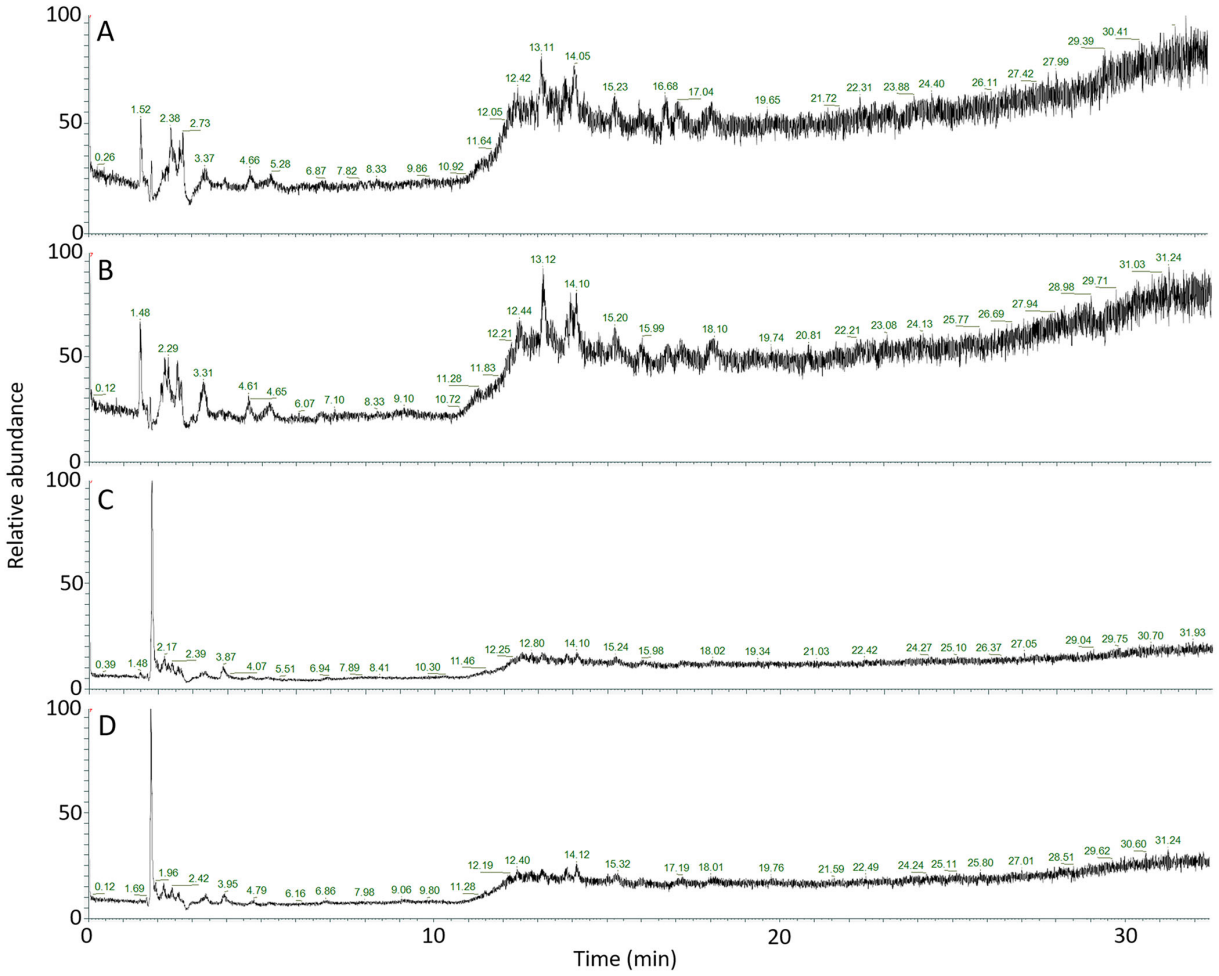
Total ion chromatograms of C1 and C2 samples for one sudden infant death syndrome (SIDS) case and one control. (A) Control sample, C1. (B) Case of SIDS sample, C1. (C) Control sample, C2. (D) Case of SIDS sample, C2. C1 and C2 total ion chromatograms differ markedly from each other, demonstrating a large effect of the type of filter card and storage conditions/time. C1; Blood prepared and stored as dried blood spot (DBS) (PerkinElmer 226 cards) for 15 years at room temperature, and C2; the same blood samples stored at −80°C for the same 15 years before preparation as DBS (Flinders Technology Associates [FTA] micro cards) shortly before analysis.

Total ion chromatograms of C1 and C2 for one SIDS case and one control are shown in Figure 3. The two C1 sample chromatograms appear visually similar, and the two C2 sample chromatograms appear visually similar. C1 and C2 sample chromatograms differ markedly from each other. Figure 3A,C represents the same whole blood sample from one control, and Figure 3B,D represents the same whole blood sample from one case of SIDS. As the chromatograms of samples with the same type of filter card (Figure 3A,B and Figure 3C,D are C1 and C2 samples, respectively) are more alike than the chromatograms originating from the same whole blood sample, this demonstrates that there is a notable effect of type of filter card and storage conditions/time on the metabolomes. The effect of these preanalytical factors appears to be more prominent than the biological differences between the samples.

### 3.3 Samples separated according to type of filter card and storage time in PCA plots

**FIGURE 4:**
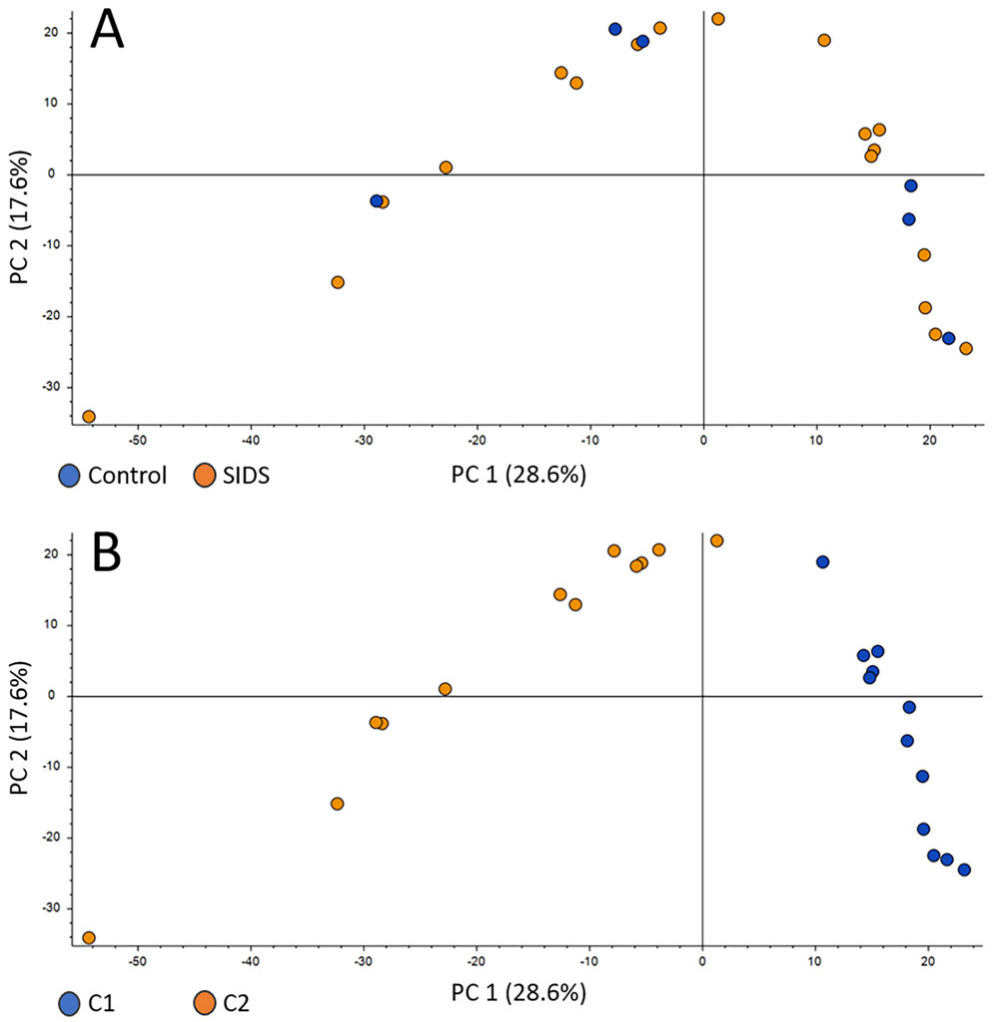
Principal component analysis (PCA) plot of nine sudden infant death syndrome (SIDS) cases and three controls with two samples per individual. (A) Colour by classification of control or case of SIDS. (B) Colour by sample category (C1 or C2). No group‐based separation between control (blue dots) and SIDS (orange dots) samples was seen (A), while a clear separation between C1 (blue dots) and C2 (orange dots) was observed (B). The effect of preanalytical factors appears to be more prominent than the biological differences between cases and controls. C1; Blood prepared and stored as dried blood spot (DBS) (PerkinElmer 226 cards) for 15 years at room temperature, and C2; the same blood samples stored at −80°C for the same 15 years before preparation as DBS (Flinders Technology Associates [FTA] micro cards) shortly before analysis.

As shown in Figure 4, samples were not separated according to classification as SIDS or control, but according to the type of filter card and storage conditions and time (PCA plots from the analysis performed in negative ionization mode are shown; the same was observed in positive ionization mode (the positive ionization PCA plot is shown Figure S1)). There was no overlap between the two groups based on PC 1 representing 28.6% of the total variation (Figure 4B). The results indicate that the effects of the type of filter card and storage conditions/time on the metabolomes at the time of analysis are much larger than that of the underlying biological differences between SIDS and controls in this study.

### 3.4 Volcano plots showed significant fold changes in almost one third of the metabolites

**FIGURE 5:**
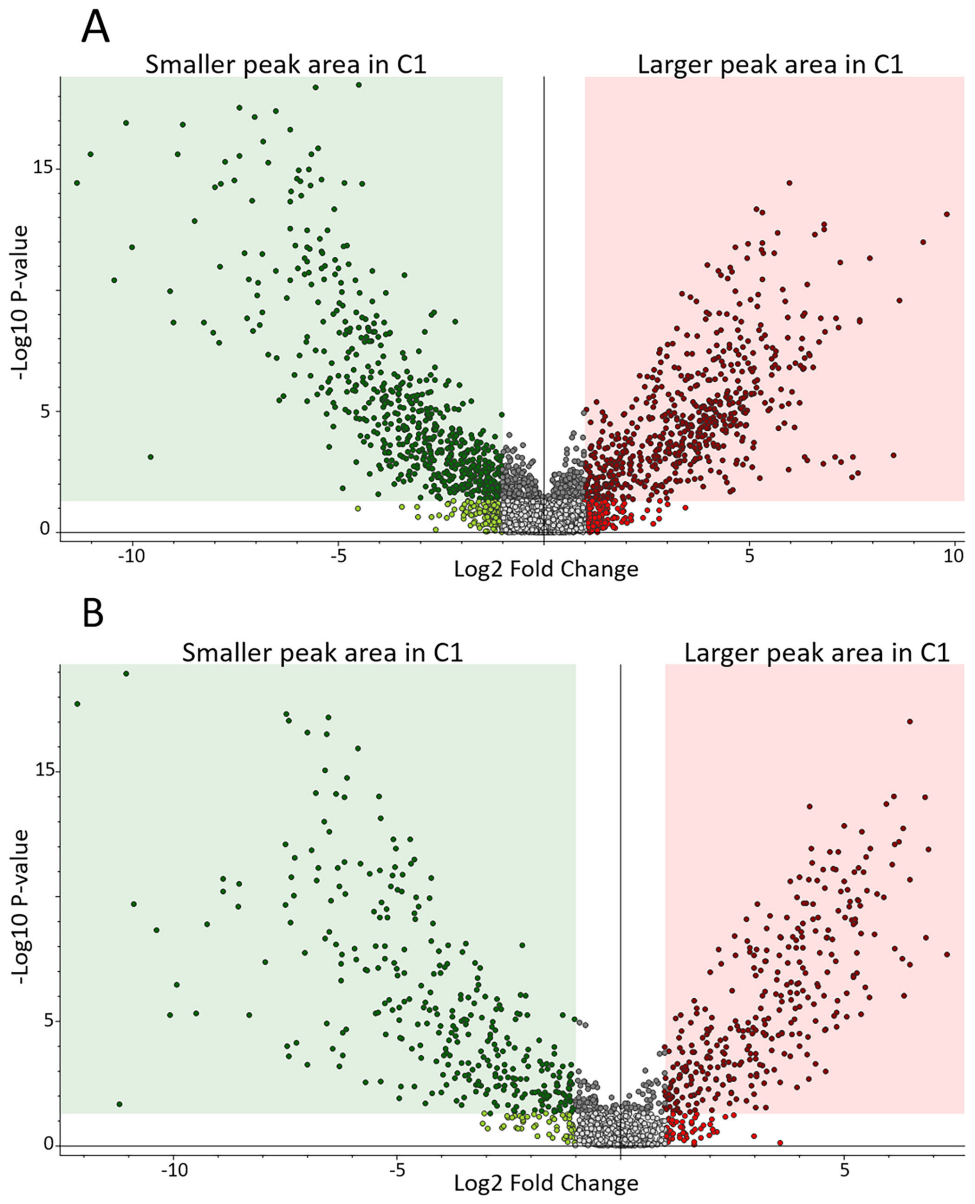
Volcano plot showing that several hundreds of metabolite features were altered, with a peak area fold change of > 2 or < 0.5 and a *p*‐value < 0.05 (i.e., metabolite features inside colored boxes), when C1 samples were compared with C2. (A) positive ionization mode. (B) negative ionization mode. Each dot represents one metabolite feature. C1; Blood prepared and stored as dried blood spot (DBS) (PerkinElmer 226 cards) for 15 years at room temperature, and C2; the same blood samples stored at −80°C for the same 15 years before preparation as DBS (Flinders Technology Associates [FTA] micro cards) shortly before analysis.

As shown in Figure 5, several hundreds of metabolites were detected with a significant 2‐fold smaller or larger peak area when C1 samples were compared with C2. For positive and negative ionization modes, respectively, 5161 and 1587 metabolite features were detected. Of these, 1263 (positive ionization mode) and 642 (negative ionization mode) met the criteria of being significantly altered with a fold change of > 2 or < 0.5 and a *p*‐value < 0.05 when comparing the two groups. In positive and negative ionization mode, respectively, 622 and 318 metabolite features were detected with a larger peak area (i.e., fold change > 2) in C1 while 641 and 324 were detected with a smaller peak area (i.e., fold change < 0.5) compared to C2. This demonstrates that a large proportion of the metabolites covered by our analysis was significantly altered when comparing C1 and C2.

### 3.5 Both accumulation and degradation of metabolites were observed

Table 2 shows a selection of the metabolites detected with a fold change of > 2 or < 0.5 and a *p*‐value < 0.05 when C1 samples were compared with C2. The metabolites selected are amongst the panel of metabolites investigated in SIDS research by Graham et al.^23^ Level of confidence^24^ for each metabolite identification is listed. Level of confidence 1 (the highest level) means that the metabolite identification is verified using our in‐house library of reference standards while level 2 means that, in addition to exact mass, the fragmentation spectrum of the metabolite matches that of an online database, also indicating a high (but not definite) degree of certainty of the identification. Lower levels of confidence (levels 3–5) do not provide definite identification at the metabolite level, but rather various degrees of information about molecular mass, molecular formula and/or possible chemical structures, etc.

**TABLE 2:** Ratio of peak areas of selected metabolites when comparing C1 samples with C2. Level of confidence 1: The metabolite identification was verified using our in‐house library of reference standards. Level of confidence 2: The exact mass and fragmentation spectrum of the metabolite matches that of an online database. C1; Blood prepared and stored as dried blood spot (DBS) (PerkinElmer 226 cards) for 15 years at room temperature, and C2; the same blood samples stored at −80°C for the same 15 years before preparation as DBS (Flinders Technology Associates [FTA] micro cards) shortly before analysis.

Compound	Level of confidence	Ratio C1/C2
Glutamine	1	0.016
Histidine	2	0.035
Ornithine	1	0.050
Proline	1	0.050
Acetylcarnitine	1	0.056
Creatine	1	0.074
Lysine	1	0.111
Serine	1	0.123
Taurine	1	0.158
Citrulline	1	0.161
Inosine‐5′‐monophosphate (IMP)	1	0.200
Threonine	1	0.232
Glutamic acid	1	0.322
Asparagine	2	0.348
Methionine sulfoxide	2	3.396
Creatinine	2	3.459
Adenine	2	50.496

**FIGURE 6:**
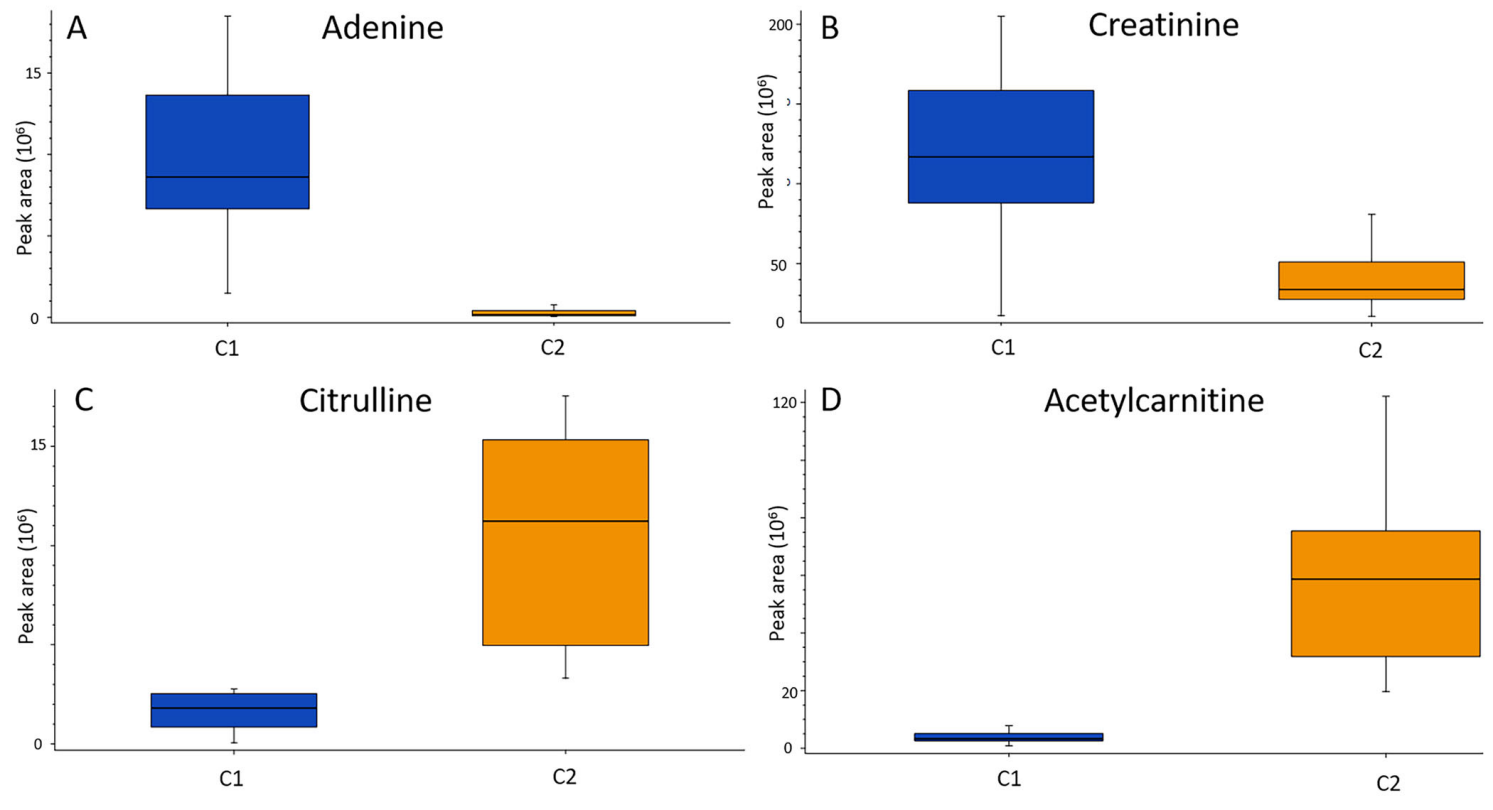
Box plots of peak areas of (A) adenine, (B) creatinine, (C) citrulline and (D) acetylcarnitine in C1 and C2 samples, demonstrating that both accumulations and degradations of metabolites were observed when comparing the two sample groups. C1; Blood prepared and stored as dried blood spot (DBS) (PerkinElmer 226 cards) for 15 years at room temperature, and C2; the same blood samples stored at −80°C for the same 15 years before preparation as DBS (Flinders Technology Associates [FTA] micro cards) shortly before analysis.

A smaller peak area was measured in C1 compared with C2 for several metabolites, including glutamine, histidine, ornithine, proline, acetylcarnitine, creatine, lysine, serine, taurine, citrulline, inosine‐5′‐monophosphate (IMP), threonine, glutamic acid and asparagine (Table 2). The opposite was found for methionine sulfoxide, creatinine, and adenine (Table 2). Figure 6 shows box plots of peak areas of selected metabolites (adenine, creatinine, citrulline and acetylcarnitine), illustrating how both an increase and a decrease in peak area was observed when comparing C1 with C2.

### 3.6 Only nicotinic acid distinguished SIDS from controls

To investigate if there were significantly altered metabolites when comparing SIDS cases with controls, we processed the freshly prepared C2 samples separately. The only significantly altered metabolite was nicotinic acid (vitamin B3), which was increased in the SIDS samples compared with the controls in the C2 group.

## 4 DISCUSSION

In this study, we demonstrated the large effects of the type of filter card on the DBS metabolome. When comparing three types of filter cards, the two chemical‐free filter cards clustered together in the PCA plot clearly separated from the FTA cards (Figure 2). Volcano plots demonstrated that the effect of filter card type alone was sufficient to significantly change the levels > 2‐fold for approximately 5% of the metabolite features detected. Although the number of samples in this pilot study was low (three samples of three healthy volunteers), the effect of filter card type was evident and illustrates that preanalytical factors can have a large effect on the metabolites measured. This was also observed for C1 and C2 in the SIDS versus controls study comparison (Figure 4); samples were clearly separated based on the type of filter card and storage and not based on clinical grouping, indicating a large effect on the metabolome according to the type of filter card and storage. This illustrates how extremely important it is to be aware of any preanalytical differences when comparing groups, and to avoid these differences in the first place whenever possible. If we in this study had not been aware that different types of filter cards were used for sampling (C1 and C2), we could have falsely assumed that the differences in the metabolomes were caused by storage differences alone. And vice versa, if we had been presented with the same samples and had been informed about the differences in filter cards, but not the differences in storage conditions (time and temperature), we would have assumed that the differences were due to filter paper type alone. In a worst case scenario, patient and control samples are sampled at different time points, using different filter cards, and handled and stored differently. Metabolite differences caused by the differences in preanalytical factors could falsely be interpreted as biological differences, e.g., biomarkers. This clearly illustrates the need for standardizing the study design a priori rather than trying to compensate for differences a posteriori.^25^

The difference in type of filter card for C1 and C2 was not part of a specific study protocol, but rather the result of a typical preanalytical difference one encounters when performing retrospective studies based on available clinical biobanks rather than properly designed prospective studies. While PerkinElmer 226 cards are chemical‐free, FTA micro cards are treated with chemicals to ensure the protection of DNA. This is known to affect the metabolomes harvested. Moretti et al. compared two types of filter cards: Whatman FTA and Whatman 903 Protein saver.^26^ The study used a phosphate buffer solution with 45 psychoactive substances which was analysed as a solution and as applied on FTA and 903 Protein saver cards. The authors found that standard deviations in the FTA analyses were lower than in the 903 analyses, and that the correlation of peak area ratios in the FTA cards and the solution was generally higher than for the 903 samples. Koster et al. investigated recovery and spot formation effects of several immunosuppressants on five different filter cards, and found Hct and concentration‐related effects for all card types, concluding that using the same type of filter card for one application is recommended.^27^ Findings in our present study strongly supports this recommendation. Of note, The U.S. Food and Drug Administration only approves two types of filter cards for human blood sampling: Whatman 903 Protein saver and PerkinElmer 226.

At the individual metabolite level, hundreds of metabolites were detected with differences in peak areas with a fold change of > 2 or < 0.5 and a *p*‐value < 0.05. For the study on healthy volunteers, around 5% of the metabolite features detected were affected by filter card type alone. For the study on SIDS versus controls, using two of the same filter cards as was used for the healthy volunteers, about one third and one fourth of the metabolites detected were affected by both filter paper and storage conditions, when comparing C1 with C2. This indicates that in addition to the effect of filter card type, significantly affecting approximately 5% of the metabolites at least 2‐fold, the effect of sample storage had an even larger impact, on an additional 20%–25% of the metabolites. Most of these metabolites are not unambiguously identified at level 1 of confidence, but they are all unique molecules (‘features’). These findings illustrate that the DBS metabolome is largely affected by preanalytical factors, emphasizing the importance of always thoroughly addressing preanalytical factors during study design and while interpreting results.

Enzymes are inactivated in DBS samples upon drying, indicating that differences between C1 and C2 samples are primarily due to non‐enzymatic reactions in samples protected from humidity.^28^ For the metabolites that were reduced in peak area as a factor of storage during long‐term DBS storage at room temperature, this can reflect low‐grade spontaneous degradation or modification over time. Vice versa, the metabolites that increase can be products of the metabolites that were degraded or otherwise modified.

Several studies have focused on preanalytical effects on biological specimens, corroborating our findings that metabolites can be largely affected by preanalytical factors. Kamlage et al. demonstrated that whole blood and plasma subjected to common preanalytical variations showed significant changes of metabolite amounts.^9^ Another study showed that after storage of DBS for 28 days at room temperature, only 55.5% of the investigated lipids were stable.^10^ In a study of DBS stored for up to 21 years at 4°C, levels of 11% of the identified metabolites (using reversed‐phase chromatography) were found to be significantly associated with storage time.^6^ Furthermore, amino acids appeared to be more unstable than other chemical classes. This is in agreement with other studies. An et al.^11^ showed significant changes of 18 out of 42 amino acids in human serum when stored for 24 h at 22°C. Han et al.^12^ highlighted the importance of storing DBS at low temperatures to increase the stability of some amino acids. Kyle et al.^29^ compared 15‐year‐old DBS stored at room temperature with matched serum samples stored at −80°C and found that lipids were more stable than polar metabolites. This is in accordance with our findings, as we found that several amino acids, as well as large amounts of other metabolites, were altered. The drug moxifloxacin was shown to be stable in DBS at room temperature for 1 month,^13^ while several fatty acids were shown to be unstable in DBS after 1 month of storage at −28°C.^14^ Drolet et al.^15^ showed that storing DBS at −20°C ensured a higher degree of stability than storing at room temperature or 37°C. Ward et al.^16^ found that after storage of DBS for one week at room temperature, almost 20% of the detected features were significantly altered. Some tools can help increase stability, e.g., a combination of an antioxidant, a chelating agent and silica gel coated paper ensured protection of omega‐3 long chain polyunsaturated fatty acids from oxidation on DBS at room temperature.^30^ It is also important to emphasize that storage time can affect DBS extraction efficiency.^31^

To ensure the stability of DBS, both −20°C and −80°C have been reported to be suitable storage temperatures.^32^ Palmer et al.^33^ reported that most of the metabolites studied were stable in DBS during storage for 12 months at −20°C and that, not surprisingly, more instability was observed when storing at 4°C or 21°C for the same period. In a protocol for the use of dried blood spots for immunoassays and molecular techniques, Grüner et al.^34^ suggest storing DBS at −20°C or lower in a zipper bag with desiccant.

In addition to the type of sample material, choice of filter card and storage conditions, there are vast numbers of other preanalytical factors often not accounted for. These include sampling techniques, procedures for application on and drying of filter cards, protection against humidity using desiccants, number of freeze/thaw cycles etc., as well as a multitude of other factors like age, gender, circadian rhythm and sleep/wake status, feeding/fasting state, medications, physical activity etc.^8^, ^25^, ^35^ These factors should be recorded and carefully considered when interpreting differences in case‐control studies in order to assess whether the differences are most likely caused by biological factors related to the disease in question, or whether the differences are due to preanalytical factors and, finally, what might be explained by random statistical variation. A particular concern related to SIDS research is the postmortem interval, as well as the storage temperature for the body prior to autopsy. Often, the abovementioned factors are not adequately addressed, and the risks of making erroneous conclusions are high. In SIDS studies and studies of other rare conditions or diseases, we must make use of the relatively few samples available, even though they are not ideally collected, handled or stored. The same goes for relevant controls. However, we must communicate the uncertainties and biases and conclude carefully when consistent study design and procedures are unobtainable. Future studies may characterize changes due to each specific preanalytical condition, but for now, our main message is that we must be careful when interpreting our results and not jump to incorrect conclusions.

We have previously evaluated the linearity of peak areas for 20 selected metabolites using one, two, three and four punches from a DBS, using the same LC‐HRMS method as in this study.^22^ R^2^ values ranged from 0.9358 to 0.9994. We also evaluated the peak area repeatability for 15 metabolites with a wide range of polarity, resulting in relative standard deviations of 2%−10%. Based on this, we feel confident that the results we present in this study represent real differences in amounts of metabolites when comparing the sample groups (chemically modified FTA cards vs. chemical‐free filter cards, and C1 vs. C2), and not random differences resulting from, e.g., technical variation.

When comparing the C2 samples separately (i.e., processing separately and comparing SIDS vs. controls), the only significantly altered metabolite was nicotinic acid (vitamin B3). Any real meaningful connection between elevated nicotinic acid and SIDS, directly or by association with other factors, is highly speculatively, and most likely is a result of random variation in a small sample group. The lack of other significantly altered metabolites could be due to the low number of samples available (only three controls and nine cases of SIDS), and the fact that SIDS can be caused by different etiologies in each individual case and that traumatic controls are equally metabolically unique, so that differences at the group level cannot be found. However, the fundamental lesson is that whatever real biological differences that were present between SIDS and controls at the time of sampling were belittled at the metabolomic level, by the differences introduced by the preanalytical effects.

### 4.1 Study limitations

One limitation of this study is the relatively low number of samples, with only 12 samples in each of the two groups C1 and C2. In spite of this, the results clearly demonstrate that the DBS metabolome is heavily affected by preanalytical factors.

In the C1 versus C2 comparison, the effect of the type of filter card and of storage time and temperature was simultaneously investigated, and the effect of each parameter cannot be specifically quantified using data from this part of the study. However, the effect of filter card type was selectively analyzed in a separate study on healthy volunteers for the exact same filter cards, thus indicating the relative effects of filter paper and storage conditions in the C1 versus C2 study. Furthermore, the main lesson is not the relative proportions of each of the dozens of possible preanalytical factors, but the realization of the fundamental importance of preanalytical factors and the need to address them both during study design and interpretation of results of metabolomics and other types of research.

Samples in this study were not corrected for Hct values, which is difficult in blood samples from the deceased due to different degrees of hemolysis. In the C1 versus C2 study, the same whole blood samples were used on both filter cards, meaning that the Hct value was the same for the two samples from each control or case of SIDS. To exemplify, one control's Hct value was e.g., 0.40 in both the C1 and the C2 sample, while one case of SIDS’ Hct value was e.g., 0.35 in both the C1 and the C2 sample. On the contrary, in the comparison of cases of SIDS versus controls, the Hct value was not the same in each group (e.g., control with a Hct value of 0.40 vs. a case of SIDS with a Hct value of 0.35), meaning that if the Hct value was known, Hct correction should ideally have been performed.

## 5 CONCLUSIONS

We have demonstrated that preanalytical differences profoundly influence metabolomics results and that without their elimination, unwarranted conclusions might be drawn and published. Real, biological differences between sample groups can be masked by the effects of preanalytical differences. To minimize the effect of preanalytical factors, our recommendation is that relevant, matched control samples ideally should be collected at the same time and stored and treated the same way as patient samples. This includes using only one type of filter card if using DBS and storage at −80°C. Ideally, to compare sample groups subject to unequal preanalytical handling, a characterization of the effect of the relevant preanalytical factors should be performed beforehand (e.g., a quantitative assessment of the effect of storage time on the relevant sample material), to enable correction for the relevant preanalytical factors.

## AUTHOR CONTRIBUTIONS

Conceptualization, H.B.S., L.F., S.H.O., T.O.R., H.R. and K.B.P.E.; Formal Analysis, H.B.S., H.R. and K.B.P.E; Investigation, H.B.S., S.R.H.W., P.O.R., L.F., S.H.O., T.O.R., H.R. and K.B.P.E.; Resources, K.B.P.E; Writing—Original Draft Preparation, H.B.S.; Writing—Review, S.R.H.W., P.O.R., L.F., S.H.O., T.O.R., H.R. and K.B.P.E.; Visualization, H.B.S.; Supervision, H.R. and K.B.P.E.; Project Administration, H.R. and K.B.P.E.

All authors have read and agreed to the submitted version of the manuscript.

## CONFLICT OF INTEREST STATEMENT

The authors declare no conflicts of interest.

## Supporting information



Supporting Information

## Data Availability

The data underlying this article will be shared on reasonable request to the corresponding author.
