# Supplementary material for: A global metabolomics minefield: Confounding effects of preanalytical factors when studying rare disorders
Source: Anal Sci Adv. 2023 Jul 21;4(7-8):255–66. doi: 10.1002/ansa.202300010 (PMC10989595; doi:10.1002/ansa.202300010)
Supplement: Supplementary file 1 — Supporting Information [file ANSA-4-255-s001.docx]

**Supporting information for:**

**A Global Metabolomics Mine Field; Confounding Effects of Preanalytical Factors when Studying Rare Disorders**

Hanne Bendiksen Skogvold^1,2^, Steven Ray Haakon Wilson^3,4^, Per Ola Rønning^1^, Linda Ferrante^5^, Siri Hauge Opdal^5^, Torleiv Ole Rognum^5,6^, Helge Rootwelt^2^, Katja Benedikte Prestø Elgstøen^2^

^1^ Department of Mechanical, Electronic and Chemical Engineering, Faculty of Technology, Art and Design, Oslo Metropolitan University, Oslo, Norway

^2^ Department of Medical Biochemistry, Oslo University Hospital, Oslo, Norway

^3^ Department of Chemistry, University of Oslo, Oslo, Norway

^4^ Hybrid Technology Hub‐Centre of Excellence, Institute of Basic Medical Sciences, Faculty of Medicine, University of Oslo, Oslo, Norway

^5^ Department of Forensic Sciences, Section of Forensic Pathology and Clinical Forensic Medicine, Oslo University Hospital, Oslo, Norway

^6^ Department of Forensic Medicine, Oslo University Hospital, Oslo, Norway

Corresponding author: Dr. Katja Benedikte Prestø Elgstøen
ORCID: 0000-0002-0087-0714

E-mail: [kelgstoe@ous-hf.no](mailto:kelgstoe@ous-hf.no)

Address: Department of Medical Biochemistry, Oslo University Hospital, Rikshospitalet, Sognsvannsveien 20, 0372 Oslo, Norway.

**Supplementary tables**

**Table S1** Settings used in MS/MS analyses in the study comparing three filter cards

| Scan range | Full MS: *m/z* 50-750  MS/MS: *m/z* 200-2000 |
| --- | --- |
| Resolution | Full MS: 70 000 MS/MS: 17 500 |
| AGC target value | Full MS: 1 000 000 ion counts  MS/MS: 500 000 ion counts |
| Maximum injection time | Full MS: 200 ms  MS/MS: 100 ms |
| Scan type | Data dependent acquisition, top 5 |
| Dynamic exclusion time | 10 s |
| Intensity threshold | 50 000 counts per second |
| Stepped normalized collision energy | 20, 50, 80 |

| Scan range | Full MS: *m/z* 50-750  MS/MS: *m/z* 200-2000 |
| --- | --- |
| Resolution | Full MS: 70 000 MS/MS: 17 500 |
| AGC target value | 1 000 000 ion counts |
| Maximum injection time | 250 ms |
| Scan type | Data dependent acquisition, top 5 |
| Dynamic exclusion time | 10 s |
| Intensity threshold | 32 000 counts per second |
| Stepped normalized collision energy | 20, 80 |

**Table S2** Settings used in MS/MS analyses of C1 and C2 samples


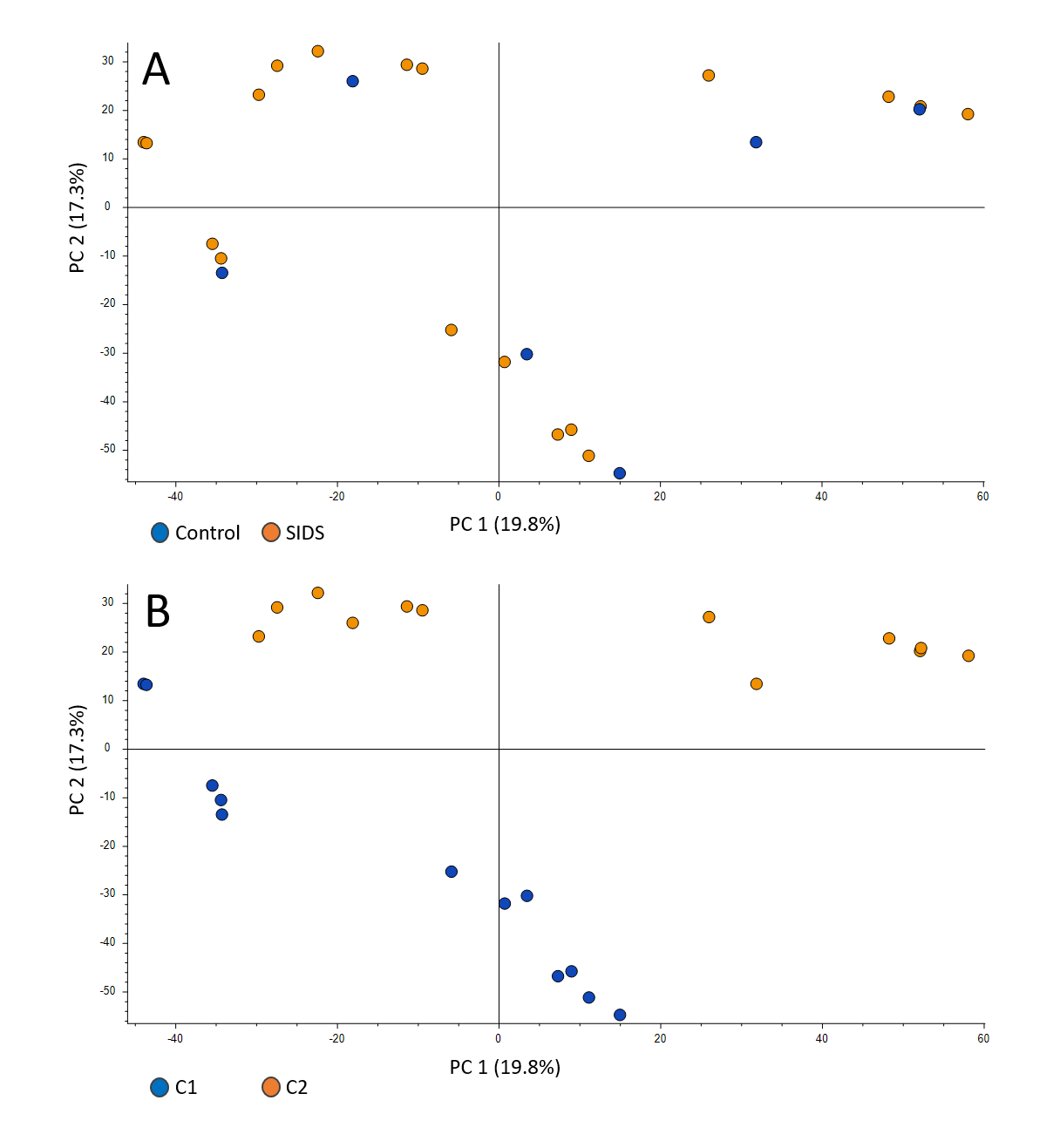


**Figure S1** PCA plot of nine SIDS cases and three controls with two samples per individual, from the analysis performed with positive ionization mode. A: colored by classification as control (blue dots) or SIDS (orange dots). No separation between the two groups is observed. B: colored by classification as C1 (blue dots) or C2 (orange dots). A clear separation between the two groups is observed along PC2. C1; Blood prepared and stored as DBS (PerkinElmer 226 cards) for 15 years at room temperature, and C2; the same blood samples stored at -80 °C for the same 15 years before preparation as DBS (FTA micro cards) shortly before analysis.
